# Clinical application of combination [^11^C]C-methionine and [^13^N]N-ammonia PET/CT in recurrent functional pituitary adenomas with negative MRI or [^18^F]F-FDG PET/CT

**DOI:** 10.1186/s12902-024-01543-2

**Published:** 2024-02-05

**Authors:** Zongming Wang, Zize Feng, Dimin Zhu, Xin Wang, Jinping Chen, Yonghong Zhu, Haijun Wang

**Affiliations:** 1 Department of Neurosurgery, Pituitary Tumor Center, the First Affiliated Hospital, Sun Yat-Sen University, Guangzhou, China; 2 Department of Neurosurgery, Jiangmen Central Hospital, Guangdong, China; 3 Department of Histology and Embryology, School of Basic Medical Sciences, Guangdong Pharmaceutical University, Guangzhou, China; 4 Department of Histology and Embryology, Zhongshan School of Medicine, Sun Yat-Sen University, Guangzhou, China

**Keywords:** Pituitary adenoma, [^11^C]C-methionine, [^13^N]N-ammonia, PET/CT

## Abstract

**Background:**

We assessed the value of positron emission tomography/computed tomography (PET/CT) with [^13^N]N-ammonia ([^13^N]N-NH3) and [^11^C]C-methionine ([^11^C]C-MET) for the evaluation and management of recurrent secreting pituitary adenoma, which could not be detected by magnetic resonance imaging (MRI) or fluorine-18 fluorodeoxyglucose ([^18^F]F-FDG) PET.

**Methods:**

Nine consecutive patients with biochemical and clinical evidence of active recurrent tumor not detected by MRI and [^18^F]F-FDG PET were enrolled in this study. All of the patients underwent [^13^N]N-NH3 and [^11^C]C-MET PET/CT, after which the pattern of tracer uptake was studied, the tumor position was located, and a clinical decision was made.

**Results:**

In general, [^11^C]C-MET had a higher uptake in pituitary adenomas (PAs) than that in pituitary tissues, while [^13^N]N-NH3 had a higher uptake in pituitary tissue than in pituitary adenomas. Increased [^11^C]C-MET uptake was observed in all nine PAs and three pituitary tissues, while all pituitary tissues and only one pituitary adenoma showed increased [^13^N]N-NH3 uptake. Four patients had concordant imaging and surgical findings indicative of biochemical remission without hypopituitarism after treatment. Radiotherapy was adopted in two patients, medication in another two, and follow-up observation in one case.

**Conclusion:**

Combined [^11^C]C-MET and [^13^N]N-NH3 PET/CT is effective in the differentiation of PAs from pituitary tissue in recurrent functional PAs with negative MRI or [^18^F]F-FDG PET. These results provide a valuable reference for further disease management.

## Background

Pituitary adenomas (PAs) account for approximately 25% of intracranial tumors [1]. PAs can be classified according to their size (microadenomas, < 1 cm; macroadenomas, ≥ 1 cm) or functional status (functional PAs that secrete endocrinal hormones and non-functional PAs that do not). Three common functional PAs, including prolactinoma secreting prolactin (PRL), acromegaly secreting growth hormone (GH), and Cushing disease secreting adrenocorticotropic hormone (ACTH), appear in approximately 75% of all PAs. Surgery is the preferred treatment for all functional PAs, except for prolactinoma sensitive to medical therapy [2]. However, recurrence rates of up to 30% have been reported [3]. The evaluation of post-surgical recurrent or residual adenoma is based on clinical symptoms, hormonal testing, and imaging analysis. Magnetic resonance image (MRI) is generally used for the diagnosis of recurrent PAs. However, it can be difficult to confirm recurrent lesions using MRI, especially recurrent microadenoma, due to post-operative changes. In clinical settings, a small number of patients may also present with clinical symptoms, hormonal hypersecretion, and negative MRI, in which the accurate localization of the lesion remains a challenge [4]. It is important to locate PAs in recurrent functional PAs using negative MRI as excess endocrinal hormones are damaging to patients’ health. Positron-emission tomography/computed tomography (PET/CT) is a powerful tool in the treatment of PAs. Fluorine-18 fluorodeoxyglucose ([^18^F]F-FDG) is the most commonly used PET imaging agent, although some scholars have reported that [^11^C]C-methionine ([^11^C]C-MET) PET can detect PAs with a higher sensitivity [5, 6]. However, [^11^C]C-MET tracers are reported to exhibit a high uptake in pituitary tissue in some patients with PAs [7, 8]. Furthermore, [^11^C]C-MET PET may incorrectly detect PAs. In addition, [^13^N]N-ammonia ([^13^N]N-NH3) was found to represent the metabolic integrity of pituitary tissue well [9, 10]. In a previous study, we found that [^13^N]N-NH3 demonstrated a higher uptake in pituitary tissue than in PAs, acting as a good tracer for normal pituitary tissue in patients with PAs [11].

In this study, the ability of combined [^11^C]C-MET and [^13^N]N-NH3 PET/CT image to effectively diagnose recurrent functional PAs and differentiate residual pituitary tissue in patients with negative MRI or [^18^F]F-FDG PET was assessed. Then, clinical decisions were made based on these results and their subsequent effects were observed.

## Methods

### Patients

Nine consecutive patients (two men, nine women) with functional PAs who had a history of transsphenoidal surgical resection more than six months half prior and pathological confirmation of secreting pituitary adenoma were included in the study. The adenomas were analyzed for categorization as PRL (*n* = 4), ACTH (*n* = 3), or GH (*n* = 2). Clinical manifestations and hormone hypersecretion were both observed post-operatively. However, 3.0-T MRI could not determine the position of the tumors. All of the patients were evaluated by PET/CT with [^13^N]N-NH3 and [^11^C]C-MET.

### PET/CT imaging

PET/CT scanning was performed using a GEMINI GXL-16 PET scanner (PHILIPS). All patients received intravenous injections of [^13^N]N-NH3 (dose range: 444–592 MBq). One day after the injection, [^11^C]C-MET (dose range: 280–450 MBq) was also injected intravenously. A 15-min emission image for [^13^N]N-NH3 or [^11^C]C-MET was obtained, beginning 5 min after the [^13^N]N-NH3 injection or 10 min after the [^11^C]C-MET injection. [^13^N]N-NH3 or [^11^C]C-MET was synthesized according to the methods described in the literature. Increased tracer uptake was in accordance with the criteria, namely that the region had a significantly higher tracer uptake than the surroundings. The pattern of tracer uptake and the maximum standard uptake value (SUVmax) were recorded and analyzed by two nuclear medicine experts.

### Analysis of the relation between imaging and treatment

Different therapeutic regimens were selected on the basis of imaging results and the type of adenoma. We compared the consistency between the intraoperative findings and the imaging results. In addition, we also studied the patients’ hormone levels before and after treatment. As a result, the reliability of imaging was assessed.

### Statistical analysis

Paired sample t-tests were used to analyze the tracer uptake levels between pituitary tissue and PAs. The data are presented as the mean ± standard deviation (SD).

## Results

**Fig. 1:**
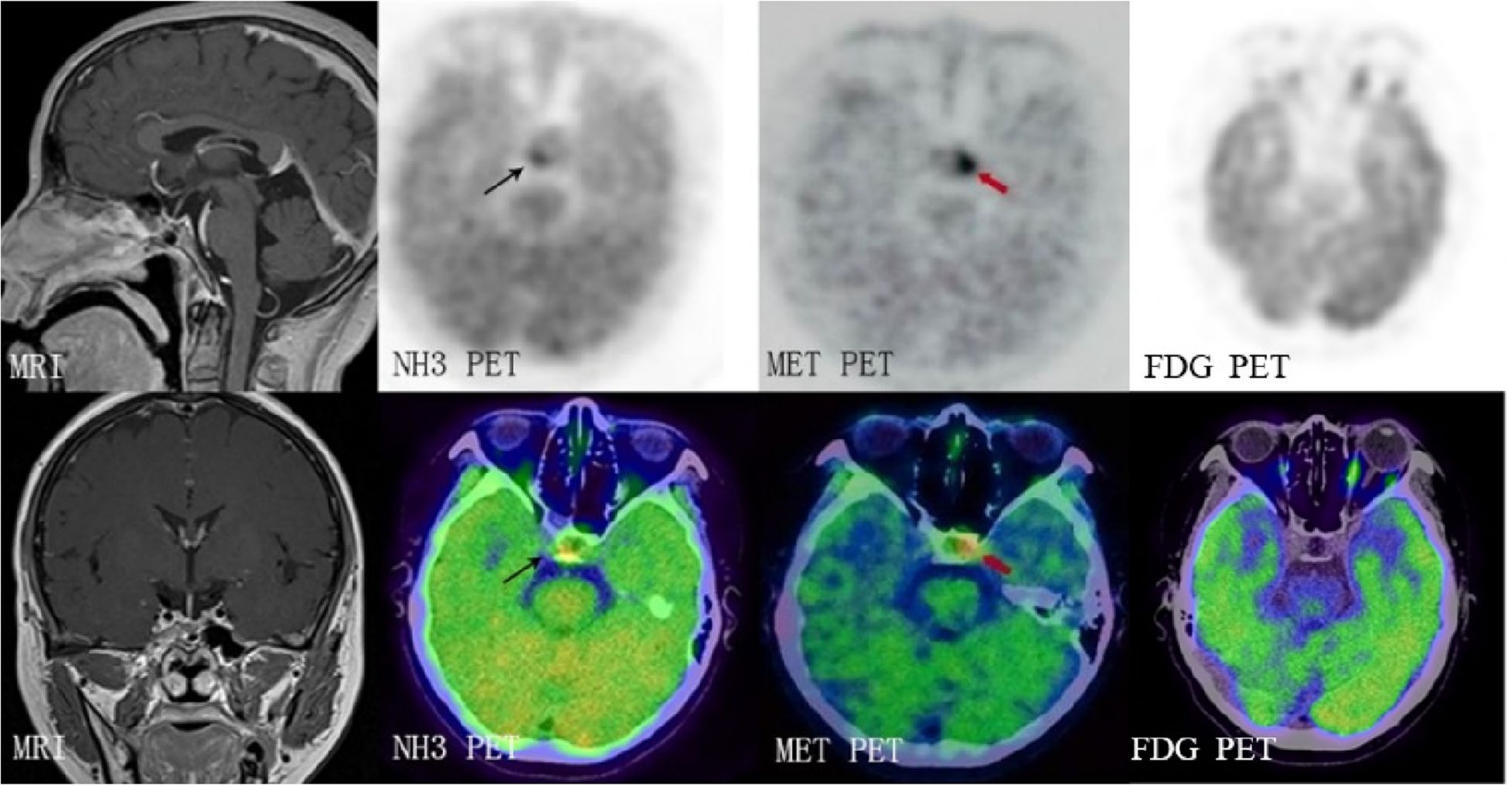
Comparison of [^11^C]C-MET to [^13^N]N-NH3 PET in the detection of residual PAs. Tumors were not detected by neither MRI nor [^18^F]F-FDG PET. [^11^C]C-MET and [^13^N]N-NH3 PET/CT images showed tumors (red arrow) and pituitary tissue (black arrow), completely differentiated by [^13^N]N-NH3 uptake and[^11^C]C-MET uptake

**Fig. 2:**
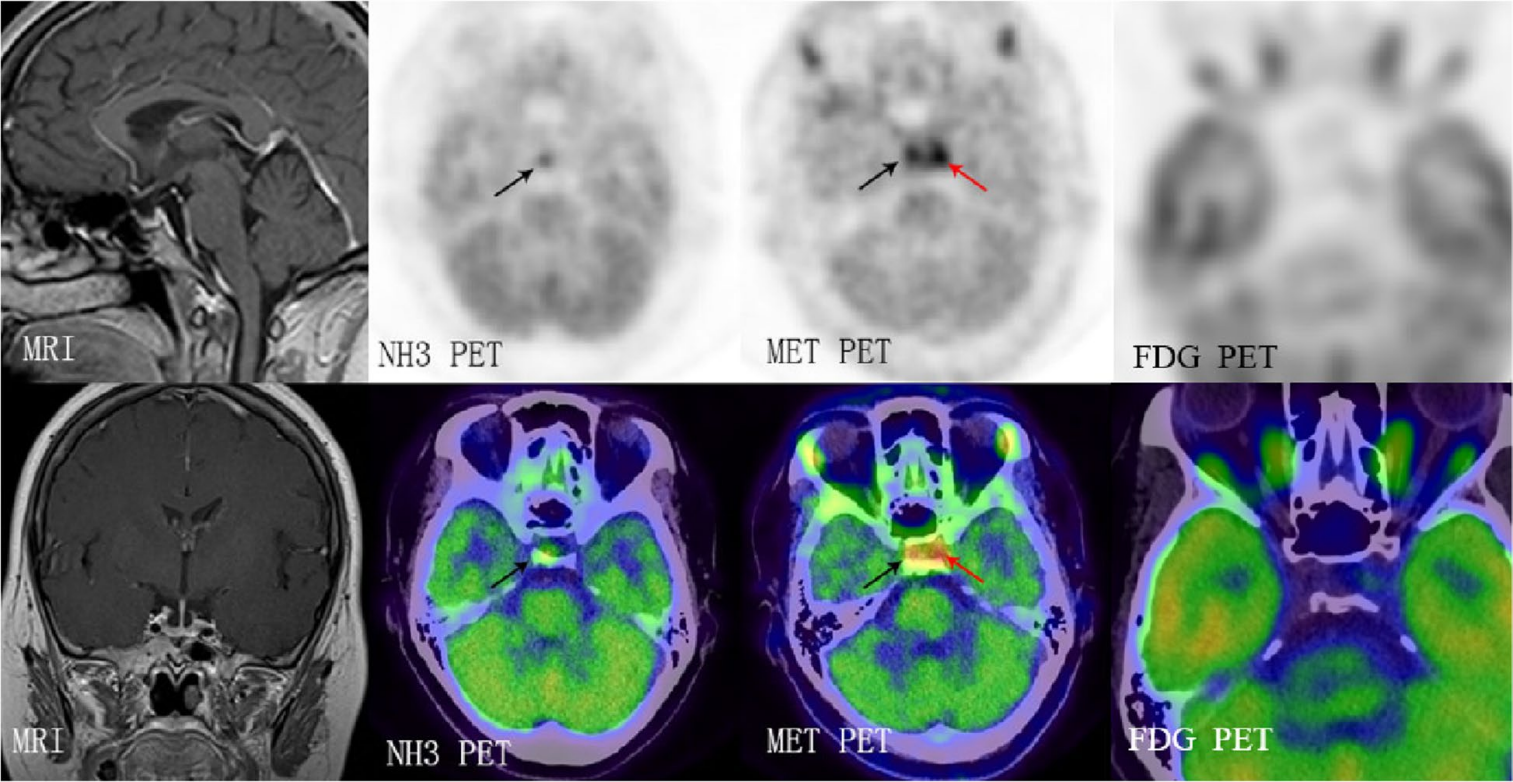
Overlap of increased [^11^C]C-MET uptake in tumors and pituitary tissue. [^11^C]C-MET PET/CT images showed that both tumors and pituitary tissue had a high [^11^C]C-MET uptake, while only pituitary tissue had a high [^13^N]N-NH3 uptake. Neither tumors nor pituitary tissue was detected by [^18^F]F-FDG PET

A summary of the SUVmax of [^11^C]C-MET and [^13^N]N-NH3 in PAs and pituitary tissue is provided in Table 1. [^11^C]C-MET uptake increased in the nine adenomas with a mean SUVmax of [^11^C]C-MET activity of 3.29 ± 0.76. Three pituitary tissues showed increased uptake of [^11^C]C-MET with a SUVmax of [^11^C]C-MET activity in all the pituitary tissue of 2.14 ± 1.29. In all pituitary tissues, increased [^13^N]N-NH3 activity was observed with a SUVmax of 2.28 ± 0.60. By contrast, only one pituitary adenoma exhibited high [^13^N]N-NH3 uptake with a SUVmax of [^13^N]N-NH3 of 1.30 ± 1.05 in all adenomas. In general, [^11^C]C-MET had a higher uptake in PAs than that in pituitary tissue, while [^13^N]N-NH3 had a higher uptake in pituitary tissue than in PAs. [^11^C]C-MET uptake overlapped in both pituitary tissue and PAs in three cases, while [^13^N]N-NH3 uptake overlapped in one case (Figs. 1 and 2).

**Table 1:** Tracer uptake in pituitary adenomas and pituitary tissue

**Patient** **No**	**secretion**	[^13^N]N-NH3 SUVmax		[^11^C]C-MET SUVmax	
		Pituitary	Adenoma	Pituitary	Adenoma
1	ACTH	2.57	1.21	1.48	3.41
2	ACTH	3.16	4.05	1.76	3.08
3	ACTH	2.38	0.85	3.66	3.93
4	GH	2.71	1.11	4.52	4.75
5	GH	1.13	0.62	2.03	3.55
6	PRL	1.75	0.83	0.92	2.03
7	PRL	2.68	1.33	2.91	3.15
8	PRL	2.15	0.78	0.88	2.95
9	PRL	1.99	0.93	1.12	2.82
Mean		2.28	1.30	2.14	3.29
SD		0.60	1.05	1.29	0.76
P		0.005		0.002

All tumors were microadenomas. Four patients agreed to undergo transsphenoidal operation again and had postoperative pathological confirmation of their PAs. During the operation, we found that the position of the pituitary tissue was consistent with [^13^N]N-NH3 PET/CT, whereas the lesions were resected under the image guidance of [^11^C]C-MET PET/CT. Four patients were estimated to be at high surgical risk because their tumors had invaded the cavernous sinus. Among these four patients, two received gamma knife therapy, while the other two were treated with medicine. The remaining patient with prolactinoma opted for careful follow-up.

Four patients treated surgically showed biochemical remission without hypopituitarism at a follow-up of one month. Two patients treated with gamma knife therapy were followed up six months after operation, and no significant change in hormone levels were observed. No difference was observed in the patient who opted for a simple follow-up. One patient treated with medicine showed biochemical remission, while another failed to attend their follow-up. The radiographic results of [^11^C]C-MET with [^13^N]N-NH3 PET/CT and patients’ outcomes are summarized in Table 2.

**Table 2:** Radiographic results of [^11^C]C-MET with [^13^N]N-NH3 PET/CT and patients’ outcomes

Patient No	secretion	Adenoma position	Pituitary position	Therapeutic decision	Follow-up Period (month)	hypopituitarism		Hormonal testing	
						**Pre-**	**Post-**	**Pre-**	**Post-**
1	ACTH	L	R	surgery	1	N	N	23.61(a)	1.22
2	ACTH	L	M	surgery	1	N	N	17.96(a)	< 0.8
3	ACTH	L	R	surgery	1	N	N	30.37(a)	< 0.8
4	GH	R&M	L	surgery	1	N	N	8.66(b)	0.93
5	GH	R^a^	L	GKRS	6	Y	Y	9.87(b)	10.32
6	PRL	L^a^	R&M	GKRS	6	N	N	267.95(c)	236.51
7	PRL	R^a^	L&M	medicine	-	Y	-	338.68(c)	-
8	PRL	R^a^	L	medicine	-	N	-	179.34(c)	15.3
9	PRL	L	R&M	observation	6	N	N	135.79(c)	127.62

(a) = serum of cortisol, (b) = serum of growth hormone, (c) = serum of prolactin,—= lost to follow-up; Reference value:(a): 2.90–19.40ug/dl, (b): 0.00 – 2.5ug/L, (c): 1.39—29.93 ng/mL

*Pre-* before treatment, *Post-* after treatment, *R* right part of sella turcica, *M* median part of sella turcica, *L* left part of sella turcica, *GKRS* gamma-knife radiosurgery, *N* no, *Y* yes

^a^localize or invaded into cavernous sinus

## Discussion

Regular follow-up was conducted after surgery for the presence of functional PAs. Although patients presented with both clinical symptoms and hormonal hypersecretion, MRI was unable to detect the presence of recurrent tumors, potentially creating confusion among physicians as to the appropriate treatment strategy to followed. Our results showed that [^13^N]N-NH3 generally showed a higher uptake in the remaining pituitary tissue than in recurrent adenomas. Furthemore, [^11^C]C-MET generally showed a higher uptake in tumors than in the pituitary tissue. [^13^N]N-NH3 PET/CT was able to better detect the remaining pituitary tissue, whereas [^11^C]C-MET PET/CT showed better results for the detection of recurrent adenomas when MRI could not. Thus, a combination of [^11^C]C-MET to [^13^N]N-NH3 PET/CT may be effective for the identification of recurrent PAs from the remaining pituitary tissue in patients with recurrent functional PAs when MRI fails, which is useful in the evaluation and management of recurrent functional PAs with negative MRI.

PET scanning serves as a supplementary tool for the detection of residual or recurrent PAs, particularly in the case of difficulties in diagnosis using MRI. Recently, several tracers, such as [^18^F]F-FDG, [^68^ Ga]Ga-DOTATATE, and [^11^C]C-MET, were applied clinically to detect tumors [6, 12, 13]. Among these, [^18^F]F-FDG and [^11^C]C-MET are the most commonly used. Adenomas may not be detected by [^18^F]F-FDG PET once activity is reduced after an operation. It has been reported that the sensitivity of [^18^F]F-FDG PET is lower than that of [^11^C]C-MET PET [7, 14]. [^11^C]C-MET PET has been reported to play a valuable role in the diagnosis of PAs with high sensitivity (86–100%) [6, 7, 15]. However, [^11^C]C-MET can be occasionally taken up by the pituitary tissue, leading to false positive results [16]. In this study, [^11^C]C-MET showed higher levels of activity in PAs than in the pituitary tissue. However, the uptake of [^11^C]C-MET by normal pituitary tissue and PAs overlapped in three cases, making it difficult to differentiate them via a single use of [^11^C]C-MET PET.

In order to avoid the false positive results of [^11^C]C-MET PET, appropriate tracer needs to be chosen with which to differentiate PAs from pituitary tissue. [^68^ Ga]Ga-DOTATATE PET has been used to detect pituitary tissue for high uptake. However, this remains an unsatisfactory approach, with one study finding that the pituitary tissue could not be revealed by [^68^ Ga]Ga-DOTATATE PET when the patient presented hypopituitarism and PA had a moderate uptake of [^68^ Ga]Ga-DOTATATE [17, 18]. [^13^N]N-NH3 is another tracer for which a higher uptake in pituitary tissue than in PAs has been reported, even with hypopituitarism, particularly in patients with a tumor diameter < 2 cm [9, 11]. In the present study, an increased uptake of [^13^N]N-NH3 was observed in the pituitary tissue of all nice patients, including two with hypopituitarism. Only one PA was found to exhibit a high uptake of [^13^N]N-NH3.

For the majority of recurrent functional pituitary adenomas, the first-line primary treatment remains transsphenoidal adenomectomy. However, if the localization of tumor is dangerous and surgical removal is difficult, other options can be selected, including treatment with medicine, radiation therapy, and chemotherapy. No matter the strategy chosen, further information is always needed to avoid damaging residual pituitary tissue and to ensure the adenoma is completely resected. In the present study, four patients underwent surgery based on the positions of adenomas and pituitary tissue obtained using PET imaging, which were consistent with the intraoperative findings and postoperative pathology. These four patients achieved biochemical remission without hypopituitarism after their operation. Two patients deemed to be at high surgical risk were administered gamma knife therapy under the guidance of the PET/CT results. Although clinical manifestations remains and no significant change was observed in the hormone levels upon postoperative follow-up after six months, we were unable to determine whether the treatment was effective due to the time interval between the gamma knife treatment and endocrine remission (ranging from 3 months to 8 years) [19]. In spite of this, obtaining further information regarding PAs and the pituitary tissue obtained through [^11^C]C-MET PET and [^13^N]N-NH3 PET may help to improve the safety of radiosurgery. However, more cases will be needed to confirm these findings.

## Conclusion

The combination of [^11^C]C-MET and [^13^N]N-NH3 PET/CT facilitates the identification of pituitary tissue and PAs in recurrent functional pituitary adenomas in cases where MRI is unable to identify the presence of recurrent tumors. Although this is a pilot clinical study, we expect these findings to help both patients in their treatment and doctors in making their clinical decisions, as well as providing a valuable reference for further studies on the treatment of recurrent tumors.

## Data Availability

The datasets used and analysed during the current study are available from the corresponding author on reasonable request.

## Abbreviations

[^13^N]N -NH3: [^13^N]N -ammonia
[^11^C]C -MET: [^11^C]C -methionine
MRI: Magnetic resonance imaging
[^18^F]F-FDG: Fluorine-18 fluorodeoxyglucose
PET: Positron emission tomography
CT: Computed tomography
PAs: Pituitary adenomas
PRL: Prolactin
GH: Growth hormone
ACTH: Adrenocorticotropic hormone
SD: Standard deviation
SUVmax: Maximum standard uptake value
[^68^ Ga]Ga-DOTATATE: Gallium-68 labeld 1, 4, 7, 10-tetraazacyclododecane-1, 4, 7, 10-tetraaceticacid-D- Phel-Tyr3-Thr8-OC
